# Solitary fibrous tumor of the floor of the mouth

**DOI:** 10.4317/jced.53492

**Published:** 2017-09-01

**Authors:** Renata-Miranda Rodrigues, Aethel-Gladys de Oliveira Fernandes, Silvia-Paula de Oliveira, Danielle-Resende Camisasca, André-Aguiar Marques, Simone-de Queiroz Chaves Lourenço

**Affiliations:** 1 DDS, MSc in Dental Clinic, Fluminense Federal University (UFF), Niterói, Brazil; 2 DDS, PhD in Oral Pathology dentist of the Federal University of Rio de Janeiro (UFRJ), University Hospital Clementino Fraga Filho (HUCFF) and Brazilian Central Army Odontoclinic; 3 DDS, PhD in oral Pathology, Department of Pathology, Dental School, Fluminense Federal University (UFF), Nova Friburgo, RJ, Brazil; Interim Professor at Espirito Santo Federal University (UFES); 4 DDS, MSc in Oral Pathology dentist of the Fluminense Federal University (UFF), Maxillofacial surgeon in the local Hospital of Itaguaí/RJ, Dentist Maxillofacial of Brazilian Army; 5 DDS, PhD in oral Pathology, Department of Oral Pathology, Professor in Odontology Graduate Program, Fluminense Federal University (UFF), Niterói, Brazil

## Abstract

**Background:**

A solitary ﬁbrous tumor (SFT) of the oral cavity is an extremely rare entity. Its diagnosis is complicated because of its diverse morphology and similarity to other mesenchymal diseases.

**Case Report:**

A rare case of SFT involving floor of the mouth is presented. The tumor was well circumscribed and almost spherical, measuring approximately 3 cm in diameter. Patient was submitted to biopsy and histopathologic examination showed a tumor composed of spindle to epithelioid cells showing pale to eosinophilic cytoplasm, oval or elongated nuclei with inconspicuous nucleoli. Tumor cells showed strong positivity for CD34, vimentin and Bcl-2. SFTs may present as a diagnostic challenge. The patient was followed for 8 years without recurrences.

**Results:**

SFT is an uncommon disease in maxillofacial region; however it should be considered in the differential diagnosis of spindle cell neoplasms with oral manifestation. Only a few cases have been reported in the floor of mouth. We describe a new case of SFT arising at this location.

** Key words:**Solitary fibrous tumors, mouth floor, oral diagnosis.

## Introduction

Solitary fibrous tumors (SFT) are unusual spindle cell neoplasms initially described in the pleura by Klemperer *et al.* 1931 (1), later on it was described in diverse extrapleural sites (2). This tumor has been found in almost every anatomic location, and both benign and malignant variants have been identified. Furthermore, with the increasing number of reported cases in a variety of extrapleural sites, it was recently suggested that extrapleural SFTs may actually develop more frequently than pleural tumors. This type of tumor occurs rarely within the oral cavity, but when reported, it is mostly found in buccal mucosa and cheek (3).

When SFT develops in oral cavity it appears like a painless swelling, some of them may give rise to compression symptoms and main clinical diagnosis include lipomas, pleomorphic adenomas, schwannomas, fibrous histiocytomas, benign glandular tumors and dermoid cyst.

Although extrapleural SFTs share the histopathologic and immunophenotypic characteristics of their pleural counterparts, because of their broad histologic spectrum, several benign and malignant spindle cell tumors can be considered in the differential diagnosis of SFT (4). When it is considered as histopathologically benign, the cellularity and collagen content characteristically differ from one area to the other. The spindle-shaped, fibroblast-like tumor cells exhibit different organization patterns and show no nuclear atypia; mitoses are rare or absent. Blood vessels are abundant (5). To establish the final diagnosis of SFT the immunohistochemical analysis is mandatory. Malignant SFTs show hypercellular lesions with moderate to marked cytological atypia, tumor necrosis and numerous mitoses and/or infiltrative margins (2). Malignant SFTs may mimic angiosarcomas and Kaposi’s sarcoma because these neoplasms are also CD34-positive but they express other endothelial markers that are typically negative in SFT (6).

The purpose of the present report is to describe a rare case of benign solitary fibrous tumor involving floor of the mouth.

## Case Report

**Figure 1:**
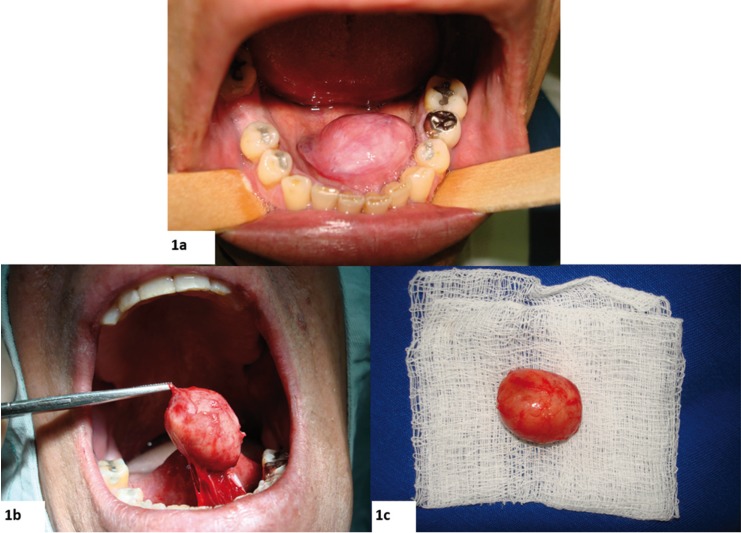
Clinical aspect of the solitary fibrous tumor in the floor mouth. (a) Clinical appearance showing a well circumscribed and almost spherical nodule; (b) Surgical resection of the lesion; (c) The surgical specimen was an oval, firm in consistency and well-encapsulated mass measuring approximately 3 cm in diameter.

**Figure 2:**
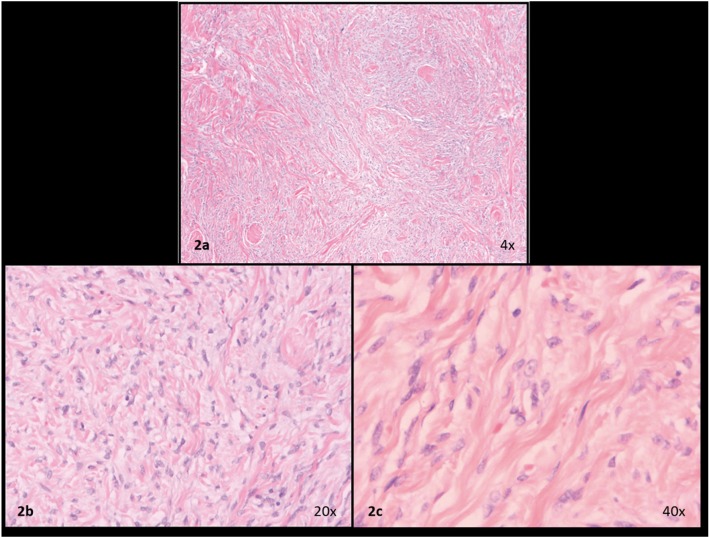
Haematoxylin and eosin stain. (a) Microscopic appearance of SFT showing a proliferation of spindle-shaped cells arranged in fascicles and in a patternless manner (H&E); (b) Highly vascularized area, with focal staghorn vessels, thin-walled and dilated blood vessels in the periphery of the tumour; (c) Collagenous tumour tissue with bland spindle-shaped and ovoid cells without atypia.

**Figure 3:**
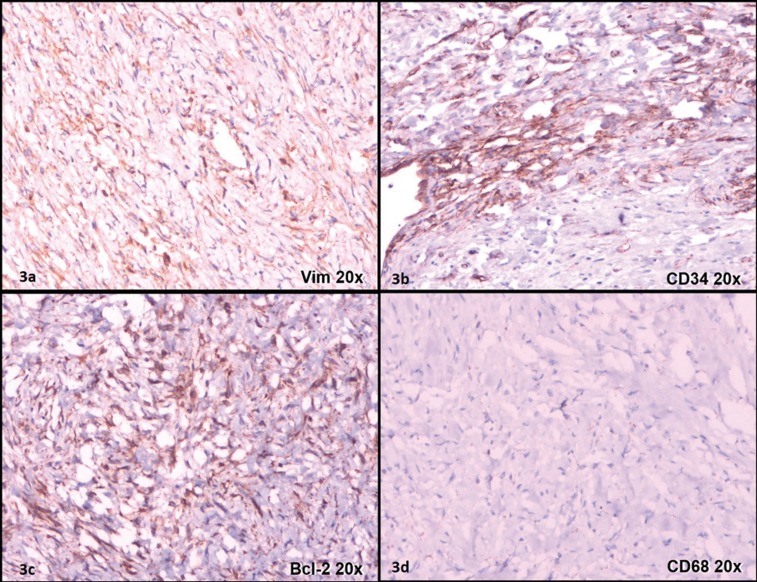
(a) Immunohistochemical staining showed strong immunopositivity for vimentin; (b) Consistent immunoreactivity for CD34 showing intense reactivity in the tumour tissue; (c) Moderate immunoreactivity for bcl-2; (d) The tumour cells are negative for CD68.

A 54-year-old Caucasian male presented at the Oral and Maxillofacial Surgery Service at Central Army Dentistry Clinic with a 14 months history of appearance of a lesion in the floor of the mouth. The patient had notice a continuous but painless increase in the size of the lesion. He declared to be a smoker and to make occasional intake of alcoholic beverages. Upon clinical examination, a firmsubmucosal mass located in the floor of mouth was detected. It was slightly displaced to the left side and was covered by normal-colored mucosa, showing discrete telangiectasias. White plaques were observed, which were suggestive of trauma from mastication. The tumor was well circumscribed and almost spherical, measuring approximately 3 cm in diameter (Fig. 1A). A clinical hypothesis of pleomorphic adenoma was raised and an excisional biopsy was performed under local anesthesia (Fig. 1B). The specimen was fixed in 10% formalin and sent for histopathologic analysis. Macroscopically, the specimen was ovoid, measured 2.8 x 2.3 x 1.4 cm, and was covered by mucosa (Fig. 1C). The cut section showed a whitish and homogeneous mass. Histologic examination showed a tumor composed of spindle to epithelioid cells (Fig. 2A) showing pale to eosinophilic cytoplasm, oval or elongated nuclei with inconspicuous nucleoli (Fig. 2B,C). Such cells were arranged in a haphazard pattern with the extracellular matrix varying from dense collagenized to loose and mixoid. The cells were arranged diffusely or in merging fascicles (Fig. 2A). Many vessels were scattered among tumor cells. They were mostly of small size, and often displayed branching lumens. There were areas of fibrosis and hyalinization (Fig. 2A). The surgical margins were free from neoplasm and a well-defined capsule was not present. Immunohistochemical stains were performed with a standard streptavidin-biotin staining method. Tumor cells showed strong positivity for CD34, vimentin and Bcl-2 (Fig. 3A, B, C). However, they were negative for CD99, desmin, S100 protein, smooth muscle actin, muscle specific actin, EMA, pan-cytokeratine and CD68 (Fig. 3D). Based on these findings the diagnosis of SFT was established. No complementary treatment was performed. The patient was followed up for 8 years without recurrences.

## Discussion

SFTs are uncommon in the maxillofacial region and they rarely originate in the oral cavity. Lesions described as being in the floor of the mouth in the English literature are shown on  Table 1. Most of them were located in the buccal mucosa, which was in accordance with SATOMI *et al.*, 2014 (3); other were in the buccal mucosa (cheek), and only five cases were in the floor of mouth.

**Table 1:**
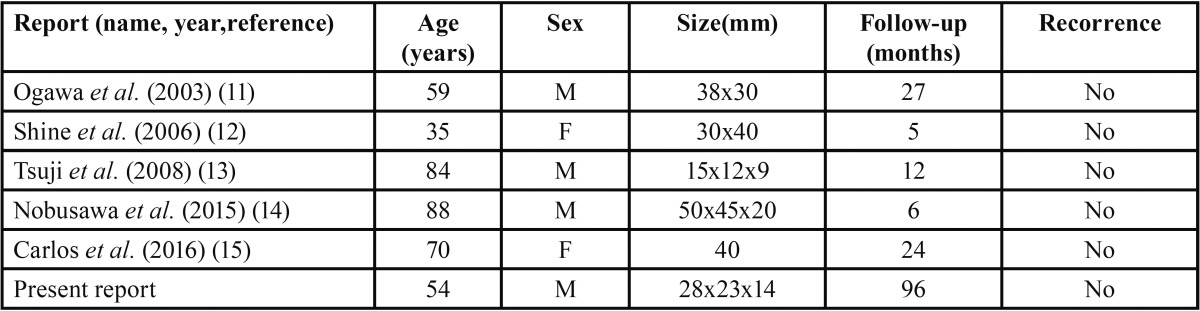
Cases of solitary fibrous tumor located in the floor of the mouth.

In the present case report, the submucosal nodule was clinically suggestive of a benign glandular tumor, such as pleomorphic adenoma, and dermoid cyst and lipoma could also be included as differential diagnosis. Spindle cell tumors were not included among the clinical diagnosis due to its rarity in this location. SFT is difficult to diagnose because of its wide morphological diversity (7). Due to the lack of any distinctive features, the final diagnosis, setting it apart from other mesenchymal tumors should be established by immunohistochemistry. SFTs have a characteristic immunohistochemical profile (8). The tumor usually shows immunoreactivity to vimentin, CD34 antigen and bcl-2 oncoprotein. CD34 antigen is widely used as an endothelial marker. Positivity for this antigen suggests either vascular endothelial origin or presence of undifferentiated mesenchymal cells. Positivity for bcl-2 oncoprotein supports a diagnosis of SFT, which is present in lymphoid tissue and which prevents cells from undergoing apoptosis. This neoplasm is negative for cytokeratins, epithelial membrane antigen, S-100 protein and endothelial markers (other than CD34 antigen), such as CD31 antigen and factor VIII-related antigen (5,7). STAT6 has been described as a specific immunohistochemical marker for SFT (14), however, we did not have access to this marker in order to use it in the present case.

In addition, differential histopathological diagnosis for SFTs may include leiomyoma, myofibroblastic inflammatory tumor (inflammatory pseudotumor), myofibroma/myofibromatosis, myoepithelioma, ﬁbrous histiocytoma, nodular fasciitis and fibroma (9). Leiomyoma in contrast to SFT, is characterized by eosinophilic spindle cells arranged more uniformly in anastomosing inter-secting fascicles, and expressing diffuse positivity for desmin and smooth muscle actin.

Inflammatory pseudotumor (inflammatory myofibroblastic tumor) consists of an admixture of spindle cells resembling SFT and aggregates of plasma cells or lymphocytes. The tumor cells, however, are usually positive for smooth muscle actin and desmin. Unlike SFT, myofibroma/myofibromatosis and fibroma are often strongly and diffusely positive for smooth muscle actin and muscle specific actin, whereas CD34 is consistently negative.

Usually, SFT is well circumscribed and partially encapsulated within the submucosa, which makes its excision easier (10). In our case the lesion was well delimitated during the biopsy, however upon microscopic analysis the capsule was not present. Maybe the absence of the capsule would require a demanding follow-up, however recurrences are rare and within 8 years of follow-up no recurrence was detected in this case (11). In most cases of SFT, complete surgical removal is curative. Prediction of behavior based on histological criteria alone is not possible. SFT at all sites may recur or metastasize after total resection in the absence or presence of atypical histological features (7). Long-term follow-up is recommended for all SFTs, due to the possibility of these lesions not being definitely benign (11).

Because of its histopathologic similarities with other soft tissue tumors, extrapleural SFT may present as a diagnostic challenge. While SFT in extrapleural sites remain uncommon, it should be considered in the differential diagnosis of spindle cell neoplasms with oral manifestation.

## References

[1] Klemperer P, Rabin CB (1931). Primary neoplasm of the pleura: A report of five cases. Arch Pathol.

[2]  Guillou  L,  Fletcher  JA,  Fletcher  CDM,  Mandaki  N (2002). Extrapleural solitary fibrous tumour and haemangiopericytoma. World Health Organization classification of tumours: pathology and genetics of tumours of soft tissue and bone.

[3] Satomi T, Hasegawa O, Abukawa H, Kohno M, Enomoto A, Chikazu D (2014). Exceptionally large solitary fibrous tumor arising from the cheek: an immunohistochemical and ultrastructural study with a review of the literature. Med Mol Morphol.

[4] Alawi F, Stratton D, Freedman PD (2001). Solitary fibrous tumor of the oral soft tissues: a clinicopathologic and immunohistochemical study of 16 cases. Am J Surg Pathol.

[5] Lukinmaa PL, Hietanen J, Warfvinge G, Sane J, Tuominen S, Henriksson V (2000). Solitary fibrous tumour of the oral cavity: clinicopathological and immunohistochemical characterization of three cases. J Oral Pathol Med.

[6] Yang XJ, Zheng JW, Ye WM, Wang YA, Zhu HG, Wang LZ (2009). Malignant solitary fibrous tumors of the head and neck: a clinicopathological study of nine consecutive patients. Oral Oncol.

[7] Sánchez-Legazaa E, Guerrero-Cauquib R, Caravallo JIM, Murga Tejada C (2011). Solitary Fibrous Tumour of the Smooth Palate. Acta Otorrinolaringol Esp.

[8] Gonzalez-Garcia R, Gil-Diez Usandizaga JL, Hyun Nam S, Rodriguez Campo FJ, Naval-Gias L (2006). Solitary fibrous tumour of the oral cavity with histological features of aggressiveness. Br J Oral Maxillofac Surg.

[9] Messa-Botero OA1, Romero-Rojas AE, Chinchilla Olaya SI, Díaz-Pérez JA, Tapias-Vargas LF (2011). Primary malignant solitary ﬁbrous tumor/hemangiopericytoma of the parotid gland. Acta Otorrinolaringol Esp.

[10] Li XM, Yu JQ, Xu GH (2014). Solitary fibrous tumor of the soft palate: A report of two cases. Oncology Letters.

[11] Ogawa I, Sato S, Kudo Y, Miyauchi M, Sugiyama M, Suei Y (2003). Solitary fibrous tumor with malignant potential arising in sublingual gland. Pathol Int.

[12] Shine N, Nor nurul Khasri M, Fitzgibbon J, O'Leary G (2006). Solitary fibrous tumor of the floor of the mouth case report and review of the literature. Ear Nose Throat J.

[13] Tsuji T, Kitada H, Abe S, Ikeda H (2008). Solitary Fibrous Tumour of the Floor of the Mouth. Asian J Oral Maxillofac Surg.

[14] Nobusawaa A, Negishia A, Sanob T, Hirato J, Oyama T, Yokoo S (2015). Solitary fibrous tumor composing benign and malignant components in the floor of the mouth: A case report. J Oral Maxillofac Surg Med Pathol.

[15] Carlos R, de Andrade BA, Canedo NH, Abrahão AC, Agostini M, de Almeida OP (2016). Clinicopathologic and immunohistochemical features of five new cases of solitary fibrous tumor of the oral cavity. Oral Surg Oral Med Oral Pathol Oral Radiol.

